# Submaximal Unilateral Arm Cycling Produces Transient but Not Sustained Changes in Corticospinal Excitability in the Homologous Muscles of the Non-Exercised Limb

**DOI:** 10.3390/brainsci16050514

**Published:** 2026-05-12

**Authors:** Hiwa Rahmani, Hamid Amoozi, Ibrahim Saif Allah Ahmed Refai, Kevin E. Power

**Affiliations:** Human Neurophysiology Lab, School of Human Kinetics and Recreation, Memorial University of Newfoundland, 230 Elizabeth Avenue, St. John’s, NL A1C 5S7, Canada; hrahmani@mun.ca (H.R.);

**Keywords:** arm cycling, transcranial magnetic stimulation, cross-education, central pattern generator, interlimb coupling

## Abstract

Purpose: This study investigated whether an acute bout of submaximal unilateral arm cycling elicits sustained changes in corticospinal excitability (CSE) and short-interval intracortical inhibition (SICI) in the homologous muscles of the non-exercised, resting limb. A secondary aim was to determine whether prior exercise induces a preconditioning effect on subsequent motor output. Methods: Transcranial magnetic stimulation was used to assess motor evoked potential (MEP) amplitude (single-pulse) and SICI (paired-pulse) in the resting non-dominant flexor carpi radialis (FCR) and extensor carpi radialis (ECR) muscles of healthy participants. Measures were obtained at rest, during a 10 min bout of unilateral arm cycling (30 W, 60 rpm), and throughout a 20 min recovery period. To assess potential preconditioning effects, measurements were repeated during a second 2 min cycling bout following a 20 min recovery. Rest and exercise conditions were analyzed separately due to differences in stimulation intensity (RMT vs. AMT). Results: Unilateral arm cycling did not produce sustained changes in CSE or SICI in the resting limb when both arms were at rest. Furthermore, unilateral arm cycling followed by a 20 min recovery period did not result in a preconditioning effect, as CSE in the resting limb was not enhanced during a subsequent unilateral arm cycling bout. Conclusions: Submaximal unilateral arm cycling induces a transient, state-dependent increase in CSE to the non-exercised limb without altering SICI. The absence of SICI modulation suggests that this facilitation is not mediated by GABA_A_-dependent intracortical mechanisms, and may instead reflect modulation arising from spinal and interlimb locomotor circuitry. The lack of sustained post-exercise effects indicates that low-intensity arm cycling does not induce a plasticity-permissive cortical state, highlighting a distinction between transient, movement-dependent facilitation and longer-lasting exercise-induced neuroplasticity.

## 1. Introduction

Among various descending pathways in motor control, the corticospinal tract plays a central role in the voluntary control of movement by transmitting motor commands from the primary motor cortex to spinal motoneurons [1]. Corticospinal excitability (CSE), which indicates the responsiveness of the corticospinal pathway to various stimuli, can be modulated by task demands, sensory feedback, and prior motor activity [2,3,4]. Such modulation of CSE could contribute to the efficiency of motor output, as increased excitability within this pathway may facilitate more effective motor unit recruitment and coordination during skilled or repetitive tasks.

Acute aerobic exercise is increasingly recognized as a potent modulator of neural excitability and short-term plasticity. Studies using lower-limb cycling have demonstrated that aerobic exercise can modulate corticospinal and intracortical excitability not only in muscles directly involved in the task, but also in remote, non-exercised muscle representations [5]. In that study, the authors showed that a single bout of moderate-intensity (65–70% of age-predicted maximum heart rate) leg cycling produced a sustained reduction in short-interval intracortical inhibition (SICI) and a concomitant increase in intracortical facilitation (ICF) in a resting upper-limb muscle [extensor carpi radialis (ECR)], with these effects persisting for at least 30 min post-exercise. There was no change in the input–output curves of CSE. These findings indicated that intracortical changes occurred in the absence of alterations in resting motor thresholds or stimulus–response curves, leading the authors to propose that aerobic exercise creates a cortical environment that is permissive to plasticity by shifting the balance between inhibitory and facilitatory intraneuronal circuits.

Arm cycling as a model of locomotion is partially generated by spinal neural networks known as central pattern generators (CPGs) [6,7]. Like lower-limb locomotor behaviors, arm cycling also engages propriospinal and commissural pathways, and has been shown to modulate CSE in active muscles in a task- [8,9], phase- [8,9] and intensity-dependent [10,11,12] manner. Arm cycling also induces crossed effects, whereby neural activity associated with the movement of one limb influences excitability in the homologous muscles of the contralateral, non-moving limb [13]. Lockyer et al. [13] examined corticospinal and spinal excitability to the biceps brachii of a resting arm while the contralateral arm engaged in submaximal arm cycling. Unexpectedly, supraspinal and spinal excitability was lowest when both arms cycled and largest when the tested arm was at rest and only the contralateral arm cycled. This observation was attributed to increased spinal motoneuronal excitability and interlimb coupling rather than purely cortical disinhibition and clearly showed an interlimb effect on measures of CSE.

Despite this, it remains unclear whether unilateral rhythmic exercise of the upper limbs is sufficient to induce exercise-related after-effects (i.e., sustained) in CSE similar to those observed following lower-limb aerobic exercise [5,14]. Specifically, it is unknown whether the neural coupling associated with arm cycling merely reflects a transient, state-dependent facilitation tied to ongoing movement, or whether it can also produce sustained effects in measures of CSE and/or cortical inhibition once the movement has ceased. Given the evidence from Singh and Staines [14] that CPG-mediated aerobic exercise can induce time-delayed changes in intracortical excitability in non-exercised, distal muscles, it is reasonable to hypothesize that arm cycling would have a similar effect on the homologous muscle of the non-exercised, contralateral limb.

Therefore, the primary purpose of the present study was to investigate whether an acute bout of submaximal unilateral arm cycling produced a lasting effect on corticospinal and/or intracortical excitability in homologous muscles of the non-exercised limb when both limbs were at rest. A secondary aim was to examine whether prior unilateral arm cycling induces a potentiation of corticospinal and/or intracortical excitability in the non-exercised limb during subsequent active cycling, reflecting a sustained facilitation of neural drive within locomotor-related circuitry. It was hypothesized that unilateral arm cycling would increase corticospinal excitability [motor evoked potential (MEP) amplitude] in the homologous muscles of the non-exercised limb [flexor carpi radialis (FCR) and extensor carpi radialis (ECR)] following the exercise bout. This facilitation was expected to be transient, progressively diminishing over time during the post-exercise recovery period and returning toward baseline levels. In contrast, SICI was not expected to change immediately following exercise, but rather to be reduced at later post-exercise time points, consistent with prior evidence suggesting a delayed modulation of intracortical inhibitory circuits following aerobic activity [5].

## 2. Materials and Methods

### 2.1. Participants

Based on prior work originating from the senior authors’ lab [13] demonstrating large modulations of MEP amplitude during arm cycling (Δ ≈ 6.3% M_max_; d ≈ 2.5), power calculations indicate that 6–8 participants would be sufficient to detect similar effects. However, given the additional variability associated with post-exercise measures, interlimb transfer, and intracortical inhibition, we conservatively estimated that 8–12 participants were required to ensure adequate statistical power. Thus, twelve young, healthy, physically active male adults participated in the study (mean ± SD: height = 174.3 ± 6.58 cm; weight = 79.00 ± 9.41 kg; age = 29.8 ± 8.4 years). The study was open to all sexes but only males volunteered. The first 12 participants that volunteered for the study were selected. Anthropometric measures and heart rate data were analyzed for all 12 participants, whereas neurophysiological analyses were performed on 11 participants due to unusable recordings from one individual. Participants were recruited from the university community and had no history of neurological disorders or upper-limb musculoskeletal injuries. A TMS Safety Screen (TMS-Screening Checklist) was used to identify any contraindications or potential risks associated with the application of TMS [15]. Hand dominance was assessed using the Edinburgh Handedness Inventory [16].

### 2.2. Experimental Setup

**Figure 1:**
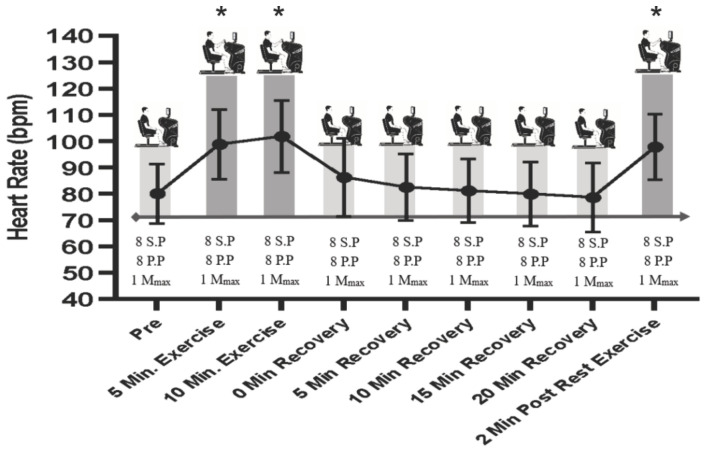
Experimental protocol and corresponding heart rate (HR) responses across time points. Schematic representation of the experimental timeline (top panels) and mean HR responses (bottom panel) measured at baseline (Pre), during exercise (5 min and 10 min), immediately post-exercise (0 min recovery), and throughout passive recovery (5, 10, 15, and 20 min), as well as during a brief re-exercise period (2 min post-rest exercise). At each time point, neurophysiological assessments were performed using single-pulse (SP) and paired-pulse (PP) stimulation protocols, with M-wave (Mmax) recorded for normalization. Exercise elicited a marked increase in HR relative to baseline, with peak values observed during the 5–10 min exercise period, followed by a progressive decline during recovery and a secondary increase during post-rest exercise. Data are presented as mean ± SD. * Significant difference from Pre and all recovery time points (*p* < 0.001). SP = single pulse; PP = paired pulse; Mmax = maximal compound muscle action potential; bpm = beats per minute.

In this experimental study design, all trials were conducted using an arm cycle ergometer (SCIFIT PRO2 Total Body Ergometer, SCIFIT, Tulsa, OK, USA). Participants were seated in an upright, comfortable position at a distance from the hand cranks that prevented trunk rotation or reaching during arm cycling (Figure 1). The seat height was adjusted so that the participants’ shoulders aligned with the ergometer’s crankshaft. The hand pedals were fixed 180 degrees out of phase, and participants were asked to wear wrist braces on both wrists during the cycling trials to limit undesirable wrist movement. Participants maintained a neutral forearm position during arm cycling. Crank positions were referenced to a clock face, with 12 o’clock representing top dead center and 6 o’clock bottom dead center.

### 2.3. Electromyography

Electromyographic (EMG) activity was recorded from FCR and ECR muscles of the non-dominant, resting arm, using Ag/AgCl surface electrodes (Kendall™ 130 foam electrodes with conductive adhesive hydrogel, Covidien IIC, Mansfield, MA, USA). In a bipolar configuration, electrodes were positioned over the mid-belly of each muscle, aligned parallel to the muscle fibers, and spaced 20 mm apart (center-to-center). The ground electrode was placed on the lateral epicondyle of the non-dominant humerus. To minimize impedance and optimize signal-to-noise ratio, the skin was shaved and gently abraded with a skin preparation gel (Nuprep Gel, Weaver and Company, Aurora, CO, USA), then cleaned with 70% isopropyl alcohol and allowed to dry before electrode placement. EMG signals were collected online at a sampling frequency of 5000 Hz using a CED 1401 interface (Cambridge Electronic Design Ltd., Cambridge, UK) and Signal 5.12 software. Signals were amplified (gain = 300) using a CED 1902 amplifier and filtered with a 3-pole Butterworth band-pass filter (10–1000 Hz).

Although our previous arm cycling studies have predominantly focused on the assessment of CSE recorded from the biceps brachii [13], our pilot testing showed that resting MEPs, especially for paired-pulse stimulation from this muscle, were difficult to obtain, particularly when both limbs were at rest. Therefore, we selected the FCR and ECR, which provided more consistent responses at rest and during tasks and have been commonly used in studies of CSE and intracortical inhibition [5,17].

### 2.4. Single- and Paired-Pulse Transcranial Magnetic Stimulation (TMS)

TMS was delivered to the motor cortex using a BiStim module connected to two Magstim 200 stimulators (Magstim, Whitland, Dyfed, UK) and a 13.5 cm outside diameter circular coil. The vertex was determined as the intersection of the midpoint of the tragus-to-tragus line and the midpoint of the nasion-to-inion line and was marked, and the coil was positioned firmly on the participant’s head, parallel to the ground [1]. The current flow was oriented to preferentially activate the dominant motor cortex.

TMS targeted the FCR and ECR muscles of the non-dominant arm, with the FCR serving as the primary muscle for motor threshold determination due to its more consistent MEP responses. The following were measured: resting motor threshold (RMT; both limbs at rest) and active motor threshold (AMT; resting threshold to the resting limb while the contralateral limb cycled). Threshold in both cases was defined as the lowest stimulation intensity required to evoke MEPs ≥ 50 µV that was clearly distinguishable from the baseline EMG in at least 4 out of 8 consecutive trials. RMT was determined while the non-dominant arm was fully relaxed. The stimulation intensity used for the remainder of the experiment was set at 120% of the respective motor threshold (RMT at rest and AMT during exercise) [13,18].

Paired-pulse TMS-evoked MEPs were recorded from the FCR and ECR muscles of the resting non-dominant arm while the dominant arm was either at rest or performing unilateral arm cycling. The conditioning stimulus (CS) intensity was determined individually for each participant, with stimulation intensity initially set based on commonly used values (~70–80% of motor threshold); however, pilot testing revealed that these intensities did not consistently produce measurable inhibition in all participants, with a flooring effect observed (test stimulus absent). Therefore, CS intensity was systematically reduced in small increments until a clear measurable, inhibitory effect on the test MEP was observed in the FCR. The selected CS intensity corresponded to the lowest intensity that reliably produced inhibition without facilitation. This resulted in CS intensities ranging between 30% and 40% of the respective motor threshold (RMT or AMT), with no values exceeding this range. The same CS intensity was then applied consistently across all experimental conditions for each participant.

Pre-stimulus EMG was also quantified to account for background activity. Consistent with previous work using arm cycling as a model of locomotor output [1], EMG values were rectified and averaged over a 50 ms window immediately preceding the stimulus artifact. These measures were collected and assessed at all experimental time points and across the nine stimulation frames within each block.

### 2.5. Brachial Plexus Stimulation (Erb’s Point)

Brachial plexus stimulation (Erb’s point stimulation) was used to measure maximal compound muscle action potential (M_max_) through supramaximal electrical stimulation of the motor nerve. A cathode and anode (Meditrace Ag–AgCl disk electrodes, 10 mm diameter; Graphic Controls Ltd., Buffalo, NY, USA) were positioned over the supraclavicular fossa and the acromion process, respectively. Stimuli were delivered as single square-wave pulses using a constant-current stimulator (model DS7AH, Digitimer Ltd., Welwyn Garden City, UK). While the dominant arm was cycling, stimulation intensity gradually increased until the M-wave amplitude plateaued, indicating M_max_ of the FCR in the resting, non-exercising hand, and supramaximal stimulation was ensured by setting the intensity at 110% of M_max_. During all recording time points, participants also received a single M_max_ stimulation.

### 2.6. Experimental Protocol

Each participant completed a single experimental session (~90 min) in the Human Neurophysiology Laboratory. AMT, RMT, and M_max_ were established at the start of the visit and used to set stimulation intensities for single- and paired-pulse TMS.

There were two conditions. In the first, CSE was assessed while participants were completely at rest. In the second, participants performed unilateral arm cycling with the dominant arm while the non-dominant arm remained at rest. Thus, regardless of the condition, the non-dominant arm was at rest throughout the study. The non-dominant hand and wrist were secured in a brace which was firmly strapped to a stable bar to standardize posture and prevent movement. One arm cycling was performed at 60 rpm and 30 W, as in our prior work [1]. TMS pulses were triggered when the cycling arm passed the 12 o’clock position, which would mean the resting limb, from which the recordings were made, would be at the 6 o’clock position if it had been cycling. This was done given the influence of synchronous vs. asynchronous arm cycling effects on CSE, which we recently demonstrated [19], due to the potential phase-dependent modulation of interlimb effects. Heart rate was monitored every 5 min using a Polar H10 heart rate sensor (Polar Electro Oy, Kempele, Finland), secured over the chest with an adjustable strap, to ensure participants exercised within a low-to-moderate aerobic intensity range and avoided high-intensity effort.

**Figure 2:**
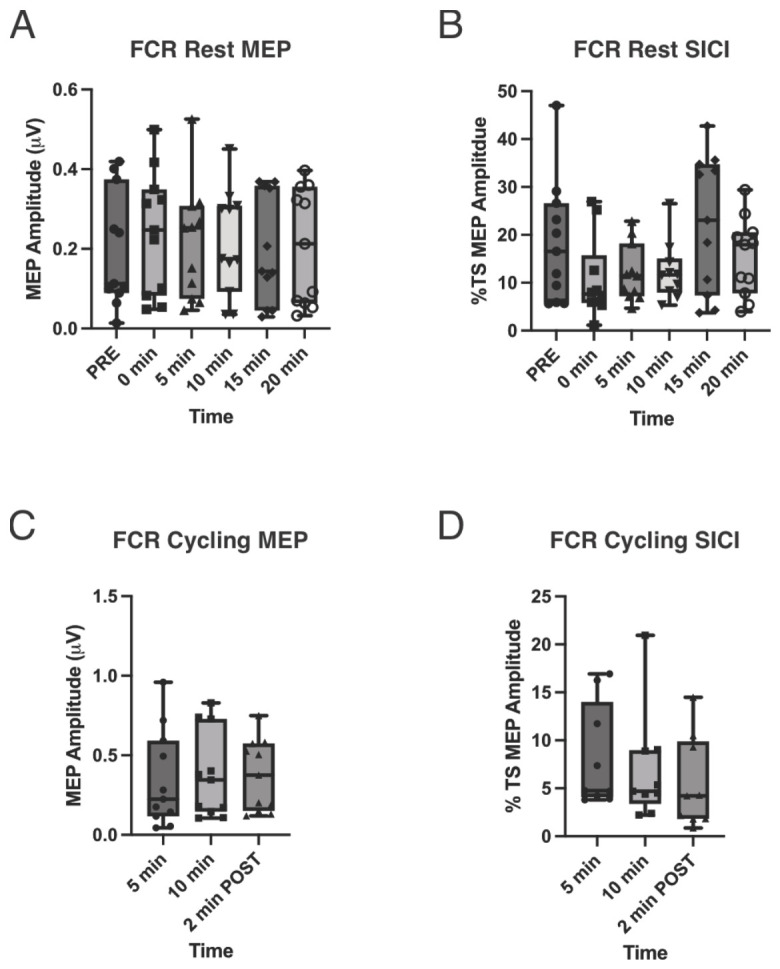
Corticospinal excitability (MEP amplitude) and short-interval intracortical inhibition (SICI) in the flexor carpi radialis (FCR) at rest and during unilateral arm cycling. (**A**) Resting motor evoked potential (MEP) amplitude (µV) measured at baseline (PRE) and at 0, 5, 10, 15, and 20 min following exercise. (**B**) Resting SICI expressed as % test stimulus (TS) MEP amplitude across the same time points. (**C**) MEP amplitude (µV) recorded during arm cycling at 5 and 10 min, and 2 min post-exercise. (**D**) SICI (% TS MEP amplitude) during arm cycling at 5 and 10 min, and 2 min post-exercise. Data are presented as box-and-whisker plots showing median, interquartile range, and full range, with individual participant data overlaid. No significant changes were observed across time for either MEP amplitude or SICI under resting or cycling conditions.

Data were collected in structured blocks throughout the experimental session. Baseline, initial exercise, and recovery phases were assessed using 5 min blocks: (i) at rest (baseline); (ii) during the initial 10 min unilateral arm cycling bout; (iii) throughout 20 min of post-exercise recovery (both arms at rest). A final cycling bout was performed as a shorter 2 min block under identical conditions. Within each block, eight paired-pulse and eight single-pulse TMSs were administered in a randomized order, and one peripheral nerve stimulation was delivered to elicit M_max_, with its trial position within the block also randomized (Figure 2).

### 2.7. Data Analysis

MEPs were analyzed by measuring the peak-to-peak amplitude of the averaged MEPs from the non-dominant FCR and ECR muscles for each trial. Peak-to-peak amplitudes were obtained using cursors in Signal software (v5.12, Cambridge Electronic Design), which were placed after the stimulus artifact and near the return of the voltage trace to baseline. The peak-to-peak amplitude of M_max_ was also assessed to monitor potential muscle fatigue and peripheral nerve excitability as a precaution, given the potential for a non-local impact of fatigue [20]. Fatigue was considered unlikely, however, given the muscles of interest were not active and that the arm cycling that was performed by the dominant limb was submaximal and used previously in our lab without causing fatigue [13]. SICI was calculated by expressing paired-pulse MEP amplitudes as a ratio to single-pulse MEP amplitudes recorded at corresponding time points.

### 2.8. Statistical Analysis

All statistical analyses were performed using SPSS (Version 29.0, IBM Corp., Armonk, NY, USA), and graphs were created using Prism (Version 10.0.3, GraphPad Software, San Diego, CA, USA). Data normality was assessed using the Shapiro–Wilk test. Because SICIs and single-pulse MEPs during rest (six time points) were determined using the resting motor threshold (RMT) and during exercise (three time points) using the active motor threshold (AMT), rest and exercise conditions were analyzed separately with repeated-measures ANOVA when data were normally distributed (*p* > 0.05) or Friedman’s tests when not (*p* < 0.05). Heart rate, recorded continuously across all nine time points using the same measurement scale, was analyzed with a single repeated-measures ANOVA. For condition-level comparisons, SICI and single-pulse MEP values were averaged across all rest and exercise time points to examine rest vs. exercise effects, with paired-samples *t*-tests applied for normally distributed data and Wilcoxon signed-rank tests for non-normal data. To evaluate potential preconditioning effects for SICI and single-pulse MEPs, values obtained at 5 and 10 min of exercise were averaged and compared with the 2 min post-rest exercise condition using parametric or non-parametric tests, depending on data distribution. Analyses were conducted separately for the FCR and ECR, with planned pairwise comparisons performed where significant main effects were observed. The significance level was set at *p* < 0.05.

## 3. Results

### 3.1. Heart Rate

A repeated-measures ANOVA was conducted to examine the effect of time on heart rate across nine time points. Mauchly’s test of sphericity indicated that the assumption of sphericity was met, χ^2^(35) = 43.35, *p* = 0.22. The analysis revealed a significant main effect of time on heart rate (F_8,88_ = 54.87, *p* < 0.001, *ηp*^2^ = 0.83). Pairwise comparisons with Bonferroni correction revealed that heart rates during the exercise phases (5th and 10th minutes of exercise and 2 min of exercise after recovery) were significantly higher than those during all recovery periods (Pre, immediately after minutes 5, 10, 15, and 20 of recovery; all *p*-values < 0.001). Importantly, there was no significant difference between exercise time points (*p* > 0.05), indicating that heart rate remained similarly elevated during all exercise phases. Also, there was no significant difference in heart rate between any of the time points when both limbs were at rest (*p* > 0.05) (Figure 1).

### 3.2. Stimulation Intensities

The stimulation intensity required to elicit a MEP differed significantly between the AMT and RMT. A paired-samples *t*-test revealed that AMT (38.7 ± 6.9% of MSO) was significantly lower than RMT (40.2 ± 7.1% MSO), t(10) = −4.28, *p* = 0.002, d = −1.29, representing a 3.7% reduction in stimulator output. This was expected and in agreement with our recent work demonstrating enhanced CSE to the homologous muscle in the inactive limb during contralateral arm cycling [13]. The CS intensity used for paired-pulse TMS averaged 38.2 ± 3.4% of RMT and 36.6 ± 3.7% of AMT (mean ± SD). A paired-samples *t*-test revealed that CS intensity was significantly lower when based on AMT compared to RMT, t(10) = 2.47, *p* = 0.033, d = 0.74. The mean stimulation intensity required to elicit the maximal compound muscle action potential (M_max_) was 103 ± 16.6 mA (mean ± SD).

### 3.3. M_max_

Maximal compound muscle action potential (M_max_) was analyzed for both FCR and ECR across all time points to assess potential changes in peripheral excitability. Repeated-measures ANOVA revealed no significant effect of time for either muscle (FCR: *F*(3.19, 31.94) = 0.29, *p* = 0.842; ECR: *F*(3.86, 38.64) = 0.87, *p* = 0.489), indicating that M_max_ remained stable throughout the experiment. The absence of changes in M_max_ suggests that peripheral nerve and muscle excitability were not altered, and that there was no evidence of fatigue, as expected, in the non-exercised limb. Given that all recordings were obtained while the target limb was at rest and that M_max_ remained unchanged, absolute MEP amplitudes were used for analysis rather than normalizing to M_max_, as normalization was not necessary under these stable peripheral conditions.

### 3.4. Single-Pulse MEPs (CSE)

**Figure 3:**
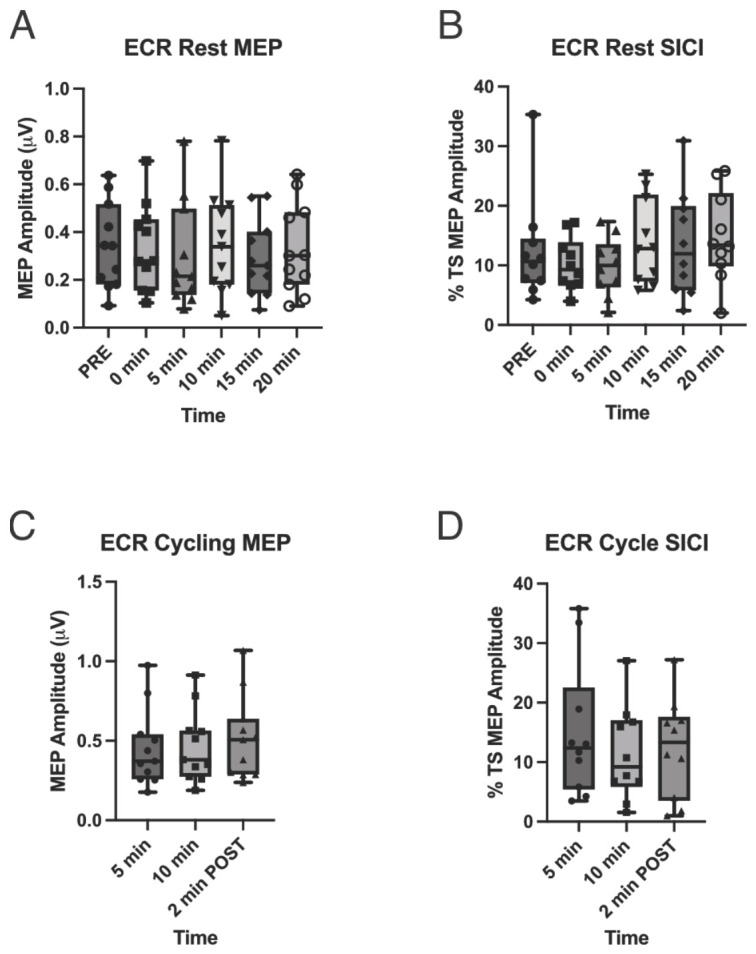
Corticospinal excitability (MEP amplitude) and short-interval intracortical inhibition (SICI) in the extensor carpi radialis (ECR) at rest and during unilateral arm cycling. (**A**) Resting motor evoked potential (MEP) amplitude (µV) measured at baseline (PRE) and at 0, 5, 10, 15, and 20 min following exercise. (**B**) Resting SICI expressed as % test stimulus (TS) MEP amplitude across the same time points. (**C**) MEP amplitude (µV) recorded during arm cycling at 5 and 10 min, and 2 min post-exercise. (**D**) SICI (% TS MEP amplitude) during arm cycling at 5 and 10 min, and 2 min post-exercise. Data are presented as box-and-whisker plots showing median, interquartile range, and full range, with individual participant data overlaid. No significant changes were observed across time for either MEP amplitude or SICI under resting or cycling conditions.

Rest: MEP amplitudes did not differ across time for FCR [F(3.89, 38.92) = 0.51, *p* = 0.72)] and had a small overall effect size (*ηp*^2^ = 0.049), indicating minimal modulation during the post-exercise recovery period relative to the pre-exercise measurement. Inspection of pairwise effect sizes relative to pre-exercise revealed only small and transient changes in MEP amplitude (0 min: d ≈ 0.30; 5 min: d ≈ 0.13; 10 min: d ≈ 0.13; 15 min: d ≈ 0.01; 20 min: d ≈ 0.06). Among the time points assessed, the largest, though still small, effect on CSE was observed immediately post-exercise (0 min) and diminished rapidly, indicating a lack of consistent or systematic modulation of CSE across time post-exercise (Figure 2A). There was no significant effect of time on ECR MEP amplitudes (F(3.58, 35.76) = 1.08, *p* = 0.378; Figure 3A).

Unilateral arm cycling: A repeated-measures ANOVA revealed no significant effect of time for either muscle (FCR: F_2,20_ = 2.49, *p* = 0.11, *ηp*^2^ = 0.20; ECR: F_2,20_ = 1.35, *p* = 0.28, *ηp*^2^ = 0.11) (Figure 2C and Figure 3C). Thus, there was no evidence of a preconditioning effect.

### 3.5. Paired-Pulse MEPs (SICI)

Rest: Due to non-normal SICI distributions in FCR and ECR (Shapiro–Wilk, *p* < 0.05), non-parametric analyses were used. Friedman’s analysis of variance by rank revealed no significant difference in SICI across the six rest time points (pre-exercise, immediately after exercise, and 5, 10, 15, and 20 min post-exercise) for either muscle (FCR: χ^2^(5) = 4.30, *p* = 0.51; ECR: χ^2^(5) = 9.38, *p* = 0.095) (Figure 2B and Figure 3B).

Unilateral arm cycling: Shapiro–Wilk tests indicated non-normal distributions of SICI for both the FCR and ECR across all exercise time points (5 and 10 min during exercise, and 2 min post-exercise) (*p* < 0.05). Friedman’s analysis of variance by rank revealed no significant difference in SICI across time points for either muscle (FCR: χ^2^(2) = 3.82, *p* = 0.16; ECR: χ^2^(2) = 0.67, *p* = 0.71) (Figure 2D and Figure 3D). Thus, there was no evidence of a preconditioning effect.

### 3.6. Pre-Stimulus EMG

The averages of the rectified pre-stimulus EMG over a 50 ms window during unilateral arm cycling were used for comparisons (5 min, 10 min, and 2 min post-rest exercise) and were compared to those recorded at rest (pre-exercise, immediately after exercise, and 5, 10, 15, and 20 min post-exercise). There was no difference in the pre-stimulus EMG in FCR (single-pulse (t(10) = −1.86, *p* = 0.067, d = −0.23) and paired-pulse (t(10) = −0.73, *p* = 0.47, d = −0.09) or ECR (single-pulse (t(10) = 1.32, *p* = 0.19, d = 0.16) and paired-pulse (t(10) = −1.80, *p* = 0.076, d = −0.22).

## 4. Discussion

The primary aim of this study was to determine whether an acute bout of submaximal unilateral arm cycling elicits sustained changes in corticospinal excitability (CSE) and short-interval intracortical inhibition (SICI) in the homologous muscles of the non-exercised limb. Contrary to our hypotheses, unilateral arm cycling did not produce sustained post-exercise increases in CSE, nor were there delayed reductions in SICI or evidence of a preconditioning effect during a subsequent bout. Although a clear increase in CSE was observed during unilateral arm cycling, this effect was transient and restricted to the period of active movement, consistent with our previous findings [13]. Importantly, direct comparisons between rest and exercise conditions were not performed due to differences in stimulation intensity (RMT vs. AMT). In contrast, SICI remained unchanged across conditions, representing a novel observation. Collectively, these results suggest that interlimb neural coupling during arm cycling reflects a state-dependent modulation of neural output rather than a lasting exercise-induced alteration in corticospinal or intracortical inhibitory processes in the non-exercised limb.

### 4.1. Crossed Corticospinal Facilitation During Rhythmic Arm Cycling

The marked increase in MEP amplitude observed during unilateral arm cycling confirms that rhythmic motor output in one limb facilitates corticospinal projections to homologous muscles in the contralateral, non-exercised limb, consistent with previous findings in proximal musculature such as the biceps brachii [13]. Importantly, the present study extends this observation to distal forearm muscles, indicating that crossed facilitation is not restricted to proximal muscle groups but reflects a more generalized increase in corticospinal excitability across the limb representation. Notably, the facilitation was similar for both flexor and extensor muscles, suggesting a global elevation in excitability rather than a reciprocal or phase-dependent modulation that typically characterizes muscles actively engaged in the cycling task. This pattern supports the interpretation that the observed effects reflect interlimb coupling mechanisms rather than task-specific motor commands directed toward the resting limb.

### 4.2. Dissociation Between Corticospinal Facilitation and Intracortical Inhibition

The dissociation between the pronounced increase in MEP amplitude and the absence of SICI modulation provides important insight into the mechanistic basis of the observed facilitation. While cross-transfer effects during high-force tonic contractions are often attributed to a release of intracortical inhibition within the primary motor cortex [21], the present findings suggest that facilitation of the non-exercised limb during rhythmic cycling is driven predominantly by mechanisms outside of the intracortical GABAergic networks probed by SICI. This interpretation is supported by the work of Lockyer et al. [13], who demonstrated that increases in MEP amplitude in a resting limb during contralateral arm cycling were paralleled by increases in cervicomedullary motor evoked potentials, indicating enhanced spinal motoneuron excitability. Together, these findings suggest that crossed facilitation during rhythmic movement may be strongly mediated by spinal mechanisms and potentially other cortical networks separate from those generating SICIs. In this context, the absence of SICI modulation implies that GABA_A_-mediated intracortical inhibitory networks were not substantially altered. Instead, the increase in MEP amplitude likely reflects subthreshold excitation of the spinal motoneuron pool via descending and propriospinal pathways. Rhythmic activity of the active limb may engage commissural and propriospinal circuits that increase the excitability of homologous motoneurons on the resting side, bringing them closer to the firing threshold. This would amplify the response to a given corticospinal volley without requiring changes in intracortical inhibition.

### 4.3. Arm Cycling Exercise Did Not Cause a Sustained Change in CSE or SICI

A main observation in this study was the rapid return of CSE to the baseline level immediately upon cessation of arm cycling. This transient profile indicates that the observed facilitation was strictly state-dependent, relying on the presence of ongoing rhythmic motor output of the contralateral limb rather than reflecting lasting use-dependent plasticity or long-term potentiation-like processes. This finding contrasts with previous work demonstrating that acute aerobic exercise can induce sustained modulation of intracortical networks [5]. Singh et al. [5] reported significant reductions in SICI and increases in intracortical facilitation (ICF) in a non-exercised upper-limb muscle following moderate-intensity lower-limb cycling, with these changes persisting for at least 30 min post-exercise. Such findings have been interpreted as evidence that aerobic exercise creates a plasticity-permissive state within the primary motor cortex. In contrast, the present results demonstrate no sustained post-exercise modulation of either CSE or SICI, indicating that the neural mechanisms engaged during unilateral arm cycling differ fundamentally from those elicited by lower-body aerobic exercise paradigms.

One potential explanation for the absence of strong interlimb effects between the arms in the present study relates to fundamental differences in the organization of upper–upper versus upper–lower limb coupling. Evidence from locomotor neurophysiology indicates that while robust bidirectional coupling exists between the arms and legs, mediated through propriospinal and spinal locomotor networks, the coupling between the two arms is comparatively weak and less functionally constrained [6,22]. In contrast to the legs, which require tightly coordinated, alternating activity to maintain balance and forward progression during bipedal locomotion, the arms are not obligatorily coupled and can operate independently to fulfill a range of task-specific functions. As a result, contralateral arm movement has minimal influence on reflex excitability and motor output in the stationary arm, whereas rhythmic leg activity exerts a strong modulatory influence on upper-limb excitability through spinal pathways. This asymmetry in coupling strength provides a mechanistic framework for the present findings: while lower-limb or whole-body rhythmic activity can facilitate corticospinal output in non-exercised upper-limb muscles, unilateral arm cycling may be insufficient to drive comparable crossed effects due to the relatively weak intrinsic coupling between the upper limbs. These findings suggest that interlimb facilitation during rhythmic movement is not uniform across limb pairings, but instead reflects the functional organization of locomotor neural circuitry [6,22].

The lack of a sustained after-effect is further evidenced by the results of the second exercise bout. Although there was a trend toward increased excitability and reduced inhibition during the second bout (preconditioning), these changes did not reach statistical significance, indicating that the initial 10 min bout of cycling was insufficient to induce a robust priming effect or lower the threshold for subsequent plasticity. This contrasts with studies demonstrating that acute aerobic exercise can facilitate subsequent plasticity or motor learning, particularly under higher intensity or longer duration conditions [23]. The rapid washout of excitability observed here suggests that the facilitation is tightly temporally locked to the execution of the movement and does not leave a sustained “memory” in the corticospinal pathway once the rhythmic drive ceases. This absence of preconditioning further supports the interpretation that the observed facilitation reflects transient, state-dependent interlimb coupling rather than an exercise-induced shift toward a plasticity-permissive cortical state.

### 4.4. Influence of Exercise Intensity

In addition to task-specific and coupling-related factors, the transient nature of the observed effects may also be attributable to the relatively low intensity of the arm cycling protocol. Exercise intensity is a key determinant of the neurophysiological response to acute aerobic exercise, with higher intensities more consistently associated with reductions in intracortical inhibition and facilitation of plasticity-related processes. The workload used in the present study (30 W at 60 rpm) was likely insufficient to generate the level of systemic and cortical activation required to downregulate GABAergic inhibition. Previous studies have shown that moderate-to-high intensity exercise can induce significant reductions in SICI and increases in ICF, potentially mediated by increased release of neurotrophic factors such as brain-derived neurotrophic factor [24]. This is consistent with work demonstrating that acute aerobic exercise can modulate corticospinal excitability in non-exercised muscles, highlighting a cross-limb effect that is sensitive to exercise characteristics such as intensity and protocol [25]. Furthermore, studies using cycling paradigms have shown that low-to-moderate intensity exercise may not consistently alter corticospinal excitability at the group level, whereas higher intensities are more likely to induce facilitation and longer-lasting neuroplastic adaptations. In contrast, low-intensity exercise tends to produce smaller or non-significant cortical adaptations. These findings support the notion of a dose–response relationship between exercise intensity and neuroplasticity, whereby a threshold level of activation must be reached to induce lasting cortical changes. While the present protocol was sufficient to engage spinal locomotor circuitry and facilitate corticospinal output during movement, it likely fell below the threshold required to induce sustained cortical disinhibition or long-term potentiation (LTP)-like plasticity.

### 4.5. Methodological Considerations

Several methodological considerations should be considered when interpreting the findings of the present study. First, pilot testing revealed that the CS intensities required to reliably elicit SICI in the forearm muscles during arm cycling, a context in which SICI has not previously been examined, were substantially lower than those used in our prior work assessing the biceps brachii during cycling and markedly lower than the CS intensities commonly reported in the literature (e.g., 70–80% RMT). When conventional CS intensities were applied, a pronounced floor effect was observed, whereby the test stimulus-evoked MEP was often abolished entirely. Under these conditions, further increases in inhibition could not be detected, precluding meaningful interpretation of task-related changes in SICI. Second, in the absence of a direct spinal measure (e.g., cervicomedullary motor evoked potentials or reflex-based indices), spinal contributions to the observed facilitation cannot be directly confirmed. However, the dissociation between robust MEP facilitation and stable SICI, combined with prior work demonstrating parallel changes in MEPs and spinal excitability during contralateral arm cycling, supports a substantial spinal contribution. Nevertheless, future studies incorporating complementary measures of spinal excitability would be required to definitively partition cortical and spinal sources of modulation. Finally, the arm cycling protocol used in this study was intentionally submaximal (30 W, 60 rpm) to isolate rhythmic motor output while minimizing fatigue and large, systemic physiological perturbations. It remains possible that higher-intensity unilateral arm cycling, longer durations, or protocols incorporating skill demands could engage additional cortical mechanisms not captured under the present conditions.

## 5. Conclusions

In conclusion, submaximal unilateral arm cycling produces a robust, yet transient, increase in CSE in the homologous muscles of the non-exercised limb. This facilitation is strictly state-dependent, emerging only during active movement and returning rapidly to baseline upon cessation. Critically, the absence of changes in SICI indicates that this effect is not mediated by GABA_A_-dependent intracortical disinhibition but instead reflects modulation arising primarily from spinal and interlimb locomotor circuitry.

## Data Availability

The data supporting the findings of this study are available from the corresponding author upon reasonable request due to ethical clearance.
